# Ultrastructural Correlates of Enhanced Norepinephrine and Neuropeptide Y Cotransmission in the Spontaneously Hypertensive Rat Brain

**DOI:** 10.1177/1759091415610115

**Published:** 2015-10-27

**Authors:** Ioannis Kourtesis, Sergey Kasparov, Paul Verkade, Anja G. Teschemacher

**Affiliations:** 1 School of Physiology & Pharmacology, University of Bristol, UK; 2 Bristol Heart Institute, University of Bristol, UK; 3 Sars International Centre for Marine Molecular Biology, University of Bergen, Norway; 4 School of Biochemistry, University of Bristol, UK; 5 Wolfson Bioimaging Facility, University of Bristol, UK

**Keywords:** hypertension, nucleus tractus solitarius, rostral ventrolateral medulla, locus coeruleus, electron microscopy, transmitter release

## Abstract

The spontaneously hypertensive rat (SHR) replicates many clinically relevant features of human essential hypertension and also exhibits behavioral symptoms of attention-deficit/hyperactivity disorder and dementia. The SHR phenotype is highly complex and cannot be explained by a single genetic or physiological mechanism. Nevertheless, numerous studies including our own work have revealed striking differences in central catecholaminergic transmission in SHR such as increased vesicular catecholamine content in the ventral brainstem. Here, we used immunolabeling followed by confocal microscopy and electron microscopy to quantify vesicle sizes and populations across three catecholaminergic brain areas—nucleus tractus solitarius and rostral ventrolateral medulla, both key regions for cardiovascular control, and the locus coeruleus. We also studied colocalization of neuropeptide Y (NPY) in norepinephrine and epinephrine-containing neurons as NPY is a common cotransmitter with central and peripheral catecholamines. We found significantly increased expression and coexpression of NPY in norepinephrine and epinephrine-positive neurons of locus coeruleus in SHR compared with Wistar rats. Ultrastructural analysis revealed immunolabeled vesicles of 150 to 650 nm in diameter (means ranging from 250 to 300 nm), which is much larger than previously reported. In locus coeruleus and rostral ventrolateral medulla, but not in nucleus tractus solitarius, of SHR, noradrenergic and adrenergic vesicles were significantly larger and showed increased NPY colocalization when compared with Wistar rats. Our morphological evidence underpins the hypothesis of hyperactivity of the noradrenergic and adrenergic system and increased norepinephrine and epinephrine and NPY cotransmission in specific brain areas in SHR. It further strengthens the argument for a prohypertensive role of C1 neurons in the rostral ventrolateral medulla as a potential causative factor for essential hypertension.

## Introduction

Central norepinephrine and epinephrine (N/E) are implicated in the regulation of a wide variety of vital brain functions in context of cognitive and emotional processing and of autonomic homeostasis. Several groups of noradrenergic and adrenergic (N/E-ergic) neurons, with cell bodies located in the brainstem and pons, project their axonal processes throughout the entire central nervous system (CNS; Moore and Bloom, 1979; Fillenz, 1990).

The largest and most densely packaged N/E-ergic cell group, the pontine area A6 or locus coeruleus (LC), provides the majority of N/E-ergic input to the forebrain, including hippocampus and cortex. The LC is associated with the control of higher central functions such as attention and vigilance, memory, decision making, and pain perception (Aston-Jones and Cohen, 2005; Sara, 2009; Carter et al., 2010; Ossipov et al., 2010; O'Donnell et al., 2012).

Brainstem N/E-ergic groups, in contrast, project predominantly to areas in midbrain, spinal cord, and within the brainstem and pons. These groups play important roles in autonomic regulation, control of energy homeostasis, and the hypothalamo-pituitary axis (Reis et al., 1984; Inoue et al., 2013). The A2 group of N/E-ergic neurons is located in the nucleus tractus solitarii (NTS) at the dorsomedial surface of the medulla oblongata and innervates hypothalamus, periaqueductal gray, amygdala, and various brainstem areas, including the rostral ventrolateral medulla (RVLM; Sawchenko and Swanson, 1982; Herbert and Saper, 1992; Aicher et al., 1996; Smith and Aston-Jones, 2008; reviewed in Rinaman, 2011). The role of the NTS in cardiovascular regulation is well documented, and A2 neurons contribute to this function (Talman et al., 1980; Colombari et al., 2001; Duale et al., 2007; Kasparov and Teschemacher, 2008). The adrenergic C1 neurons in the RVLM innervate nuclei in the pons and diencephalon and also the thoracic spinal cord (Ross et al., 1984a; Milner et al., 1989; Verberne et al., 1999; Card et al., 2006). C1 activity is essential for regulation of autonomic and cardiovascular functions, and altered signaling within RVLM contributes to neurogenic hypertension and progression of heart failure (Ross et al., 1984b; Colombari et al., 2001; Guyenet, 2006; Kasparov and Teschemacher, 2008; Marina et al., 2013). Recent experiments with optogenetic activation of C1 neurones in the RVLM have further implicated this cell group in a variety of other physiological processes including arousal and breathing (Burke et al., 2014; Abbott et al., 2014).

Cotransmission is a common feature of peripheral and central N/E-ergic neurons (Teschemacher and Johnson, 2009). For example, although C1 neurons in the RVLM synthesize and release catecholamines, these neurons are also equipped with the machinery for glutamatergic signaling (Teschemacher et al., 2008; Burke et al., 2014; Abbott et al., 2014). In addition, many studies have documented that, in the periphery as well as in the brain, N/E-ergic neurones frequently coexpress neuropeptide Y (NPY; Lundberg et al., 1982, 1983; Everitt et al., 1984; Sawchenko et al., 1985; Holets et al., 1988; Wilcox and Unnerstall, 1990; Zardetto-Smith and Gray, 1995). Moreover, it is well established that NPY can be coreleased with N/E in the periphery (Lundberg et al., 1986; Pernow, 1988; De Potter et al., 1997; Johnson, 2010) and that cotransmission modulates N/E signaling in context of blood pressure control (Chalmers et al., 1989; Zukowska-Grojec et al., 1993; Shanks and Herring, 2013). A mouse model in which NPY is overexpressed in N/E neurones globally has elevated sympathetic activity and is prone to stress-induced hypertension, supporting the idea that NPY contained within N/E-ergic fibers plays a role in sympathetic drive (Ruohonen et al., 2009). Within the CNS, NPY also influences cardiovascular regulation, but the effects are mediated via multiple pathways and may be sympatho-excitatory or -inhibitory, depending on the brain area NPY is acting upon (Vallejo and Lightman, 1986; Fuxe et al., 1987; Fuxe et al., 1990). A clear example for central N/E–NPY cotransmission was demonstrated in a study where the RVLM was stimulated, causing not only an increase in blood pressure via the descending excitation of spinal preganglionic sympathetic neurons but also release of NPY immunoreactivity into the subarachnoid space (Morris et al., 1987).

There is a consensus that N/E in the brain are predominantly released from nonjunctional varicosities and modulate regional circuitry via volume transmission (O'Donnell et al., 2012). An increasing body of evidence supports the idea that astrocytes are a primary target for central N/E release and essential for mediating modulation of synaptic transmission in healthy brain function (Hertz et al., 2004; Pankratov and Lalo, 2015). The volume transmission mode calls for N/E storage in large dense core vesicles (LDCV) located outside of presynaptic zones (Trueta and De-Miguel, 2012). Although previous electron microscopic (EM) studies ascribed two distinct vesicle populations to central N/E-ergic neurons, small clear vesicles (diameter range 40–60 nm) and LDCV (80–120 nm), the evidence for functional N/E storage indeed appeared stronger for the LDCV population (Bloom and Aghajanian, 1968; Pickel et al., 1989; Nirenberg et al., 1995). The first direct evidence that this type of release actually exists in the brain came from our microamperometric recordings, which demonstrated rare but very large quantal release events in central N/E-ergic neurons (Chiti and Teschemacher, 2007), which could not be easily explained by the earlier EM observations. However, a comparative and quantitative analysis of N/E-containing vesicular populations across several N/E-ergic nuclei of the brain has never been performed. Moreover, it was never tested whether a pathological phenotype associated with altered central N/E-ergic transmission may be associated with ultrastructural changes in the N/E-ergic vesicular populations.

An obvious pathology to focus on in the context of increased central N/E activity is neurogenic hypertension. We therefore chose to draw comparisons between a control rat model, the Wistar rat (WR) and the spontaneously hypertensive rat (SHR), which shows a complex phenotype consistent with overactivity/overreactivity in central N/E transmission. Not only is this strain a commonly used model for studying essential hypertension (Frohlich, 1986; Kasparov and Teschemacher, 2008; Xu et al., 2012), but it also carries traits consistent with features of attention-deficit/hyperactivity disorder (ADHD; Sagvolden, 2000; Sontag et al., 2013) and vascular dementia (Sabbatini et al., 2002). Using microamperometry, we demonstrated earlier that in SHR, there is a major shift toward much larger N/E quantal release events in the RVLM, but up to now there was no known morphological correlate for these surprisingly large release events.

Given the paucity of information about the types of N/E-containing vesicles in the brain and their costorage of NPY, we decided to undertake an EM study using more advanced methods of tissue fixation and immunolabeling, which have been developed specifically by others and ourselves to preserve fragile vesicular structures (McDonald et al., 2007; Verkade, 2008). We performed immuno-gold labeling of dopamine-β-hydroxylase (DbH) and vesicular monoamine transporter 2 (VMAT2) with validated antibodies at the ultrastructural level. By combining light fluorescent microscopy and EM, we have obtained by far the greatest sample of measurements of N/E-ergic vesicles in mammalian CNS to date. The aim of the current study was twofold. Our first objective was to map NPY coexpression in central N/E-rgic neurons relevant to hypertension and ADHD, based on three of the most relevant N/E-ergic groups: A6 in the LC, A2 in the NTS, and C1 in the RVLM. Second, we intended to carefully evaluate the N/E containing vesicular structures in these groups, in order to get a clear view of their sizes and NPY content and any differences between the WR and the SHR strains.

## Materials and Methods

### Animals

Animal experiments were carried out in accordance with the U.K. Animals (Scientific Procedures) Act 1986, consistent with the Guide for the Care and Use of Laboratory Animals published by the U.S. National Institutes of Health (NIH Publication, 8th Edition, 2011), and approved by the University of Bristol Ethical Review Group (UB/05/035). Animals were group-housed an enriched environment under a standard 12-hr light/dark cycle, with free access to food and water. Experiments were conducted in nine adult male WR and SHR each (250–310 g, Harlan Laboratories, UK), with three animals of each strain allocated to confocal microscopy and, to EM analysis, three animals each for brainstem preparations and for LC, respectively.

### Immuno-Fluorescence Confocal Microscopy

Animals were terminally anesthetized using pentobarbital (400 mg/kg i.p.) and perfusion-fixed transcardially with 4% paraformaldehyde. Sections of 40 μm were prepared from the brainstem and pons regions on a freezing microtome. Free floating sections (40 μm) were placed in blocking solution for 1 to 2 hr (3% goat serum; 1–2% bovine serum albumin (BSA); 0.1% phosphate buffered solution [PBS]-Triton X-100) at room temperature and subsequently incubated with primary antibodies (1:200 DbH rabbit polyclonal, Abcam AB43868; 1:400 NPY mouse monoclonal, kind gift of Dr. Grouzmann, Lausanne) in the blocking solution for 16 to 24 hr.

The DbH antiserum was previously characterized in Western blots, as well as by immunohistochemistry studies, where it selectively labeled N/E-ergic neurons, and, at a subcellular level, both secretory vesicle lumen and vesicle membranes (Nosjean et al., 2002; Fortune and Lurie, 2009; Polanski et al., 2010). In our hands, it also reliably labeled central N/E-ergic neurons in all established locations. The NPY antiserum provided by Dr. Grouzmann belongs to the immunoglobulin G1 subclass with high affinity to the 11 to 24 amino acid epitope region and with no cross-reactivity for peptide YY and pancreatic polypeptide (Grouzmann et al., 1992).

Sections were then incubated in Alexa Fluor anti-rabbit-594 (Invitrogen) and Biotin anti-mouse (Vector), followed by Streptavidin-Alexa Fluor 488-conjugated antibody (Invitrogen) for 1 to 2 hr, respectively. Negative controls were performed by omitting the primary antibody. FluorSave (CalBiochem) was used to mount sections. Fluorescent images were acquired with a confocal laser scanning microscope (Leica SP1, Leica Microsystems, Wetzlar, Germany). For each animal, approximately 66 brainstem sections containing NTS (44 rostral and 22 caudal, relative to the area postrema), 40 sections for caudal ventrolateral medulla, 33 sections for RVLM, and 20 pontine sections containing LC were evaluated.

### AVV Transduction

Tissue for EM processing was selected from N/E-ergic neuron-dense areas in NTS, RVLM, and LC, which were preselected by specific expression of enhanced green fluorescent protein under the control of the PRS×8 promoter using adenoviral vector (AVV) transgenesis (Hwang et al., 2001; Lonergan et al., 2005; Teschemacher et al., 2005). The approach has the advantage of labeling living neurones, allowing us to rapidly localize and preselect N/E-ergic areas for EM sample preparation. This procedure minimized pre-EM tissue damage and profoundly increased our data collection efficiency, thus yielding sufficiently large sample sizes to carry out quantitative analysis. Diameters of control vesicles detected in untransduced tissue were consistent with the peak range in transduced brains. Transgenic expression of enhanced green fluorescent protein in neurons is a widely used approach, and this AVV has been extensively characterized by us and others and causes no appreciable changes in central N/E-ergic transmission (Lonergan et al., 2005; Teschemacher et al., 2005; Teschemacher et al., 2008; Abbott et al., 2009). For viral vector injection, animals were anesthetized with ketamine/medetomidine (60/250 mg/kg, i.m.) and positioned in a stereotaxic frame. Core temperature was maintained at 37 ℃. AVV was injected at a titer of 10^10^ TU/ml, 0.5 μl per site injection, at a rate of 0.5 µl/min. For the NTS, three bilateral microinjections of AVV were made along the length of the NTS at bregma −14.30, −14.60, and −13.80. For the RVLM, a unilateral injection of AVV was performed at bregma between −11.3 mm and −12.3 mm. In the LC, four unilateral AVV injections were placed at bregma −9.2 mm, −9.7 mm, −10 mm, and −10.5 mm. At the end of surgery, anesthesia was reversed with atipamezole (1 mg/kg).

### Electron Microscopy Sample Preparation

A combination of perfusion- and cryo-fixation with resin embedding was used to achieve an optimized balance between high labeling efficiency as obtained by the Tokuyasu cryo immuno-gold labeling technique and ultrastructural visualization as obtained by standard Epon embedding (van Lookeren et al., 1991; Verkade et al., 1995). Twelve to fifteen days following AVV transduction, the animals were sacrificed with an overdose of pentobarbital (400 mg/kg, i.p.) and perfusion-fixed transcardially with 4% paraformaldehyde in PBS. Brain sections of 200 µm were sliced with a vibratome (Camden Instruments, Loughborough, UK), and green fluorescent N/E-ergic cells were identified under a fluorescence microscope (Leica DM IL LED; Leica Microsystems). A tissue puncher (Leica) was used to extract 1-mm circular tissue blocks that were incubated in cryoprotectant (20% bovine serum albumin, 100 mM trehalose in PBS), loaded into a 0.2-mm deep carbon-coated membrane carrier (Leica Microsystems), and subjected to (virtually instantaneous) high pressure freezing (EM PACT2 + RTS; Leica Microsystems; Verkade, 2008). Samples were subsequently freeze-substituted (AFS2 + FSP, Leica Microsystems) and embedded in Lowicryl HM20 as previously described (van Weering et al., 2010). Ultrathin 100-nm sections were sliced (Reichert Ultracut E) and placed on Pioloform-coated copper slot grids (2 × 1 mm; Agar).

### Ultrastructural Immuno-Gold Labeling

Grids were placed on 100 mM glycine in PBS to quench aldehyde and then in 0.1% BSA-c™ (Aurion, Wageningen, the Netherlands) in PBS (blocking solution). This was followed by incubation of alternating sections in primary antibodies of either of the two N/E-ergic markers, DbH (1:20 rabbit polyclonal; Abcam, see earlier) or VMAT2 (1:40 rabbit polyclonal; Millipore AB1598P) and NPY (1:40 mouse monoclonal; kind gift of Dr. Grouzmann as described earlier), in blocking solution for 1 hr. The specificity of the VMAT2 antiserum (rabbit polyclonal; Millipore AB1598P) had been previously established by multiple Western blot and immunohistochemistry studies, including subcellular distribution using electron microscopy (Liu et al., 1994; Peter et al., 1995; Nirenberg et al., 1995). The specificity was confirmed by substitution of the primary antibody with nonimmune serum or by preadsorption of the primary antibody with the peptide antigen. In our study, the antiserum labeled the membrane of LDCV, following a pattern consistent with DbH immunolabeling and in an identical pattern to that described by previous work in both rat brain tissue and the adrenal gland (Peter et al., 1995).

Sections were subsequently labeled with secondary antibodies (10-nm colloidal gold donkey antirabbit for DbH/VMAT2 and 15-nm colloidal gold goat antimouse for NPY; Aurion) in blocking solution for 45 to 60 min. Sections were then counterstained in 3% uranyl acetate and lead citrate. Images of the ultrastructure and immuno-gold labeling were acquired on a Tecnai12 Biotwin transmission electron microscope equipped with a bottom-mount 4 × 4*k* CCD camera (FEI company, Eindhoven, the Netherlands). Immunolabeling was optimized to achieve efficient labeling of N/E-ergic vesicles while minimizing nonspecific gold staining, which was confirmed by the absence of gold particles from structures renowned for background labeling, such as nuclei, mitochondria, and myelin sheaths. Negative controls were performed by omitting primary antibodies, which resulted in the absence of gold labeling in all brain areas examined.

EM image analysis was performed on four to six sections per resin block per rat (*n* = 3) for each brain region. This approach yielded statistically significant outcomes and allowed consistent random sampling of the ultrastructure and antigenicity representative for each investigated brain region. The horizontal and vertical radii of vesicles were measured in Fiji/ImageJ, and they were averaged for the purpose of further evaluation. DbH and VMAT2 labeling appeared in a consistent pattern of dense core vesicles, with VMAT2 labels observed associated with vesicular membranes and DbH labeling seen at either membrane or lumen of the vesicle. The size distributions and number of vesicles labeled by the two markers were alike and thus pooled for analysis.

Statistical difference between vesicle distributions belonging to different brain areas in either WR of SHR was studied by application of the two-sample Kolmogorov–Smirnov (KS) test (http://www.physics.csbsju.edu/stats/KS-test.n.plot_form.html).

### Mathematical Model for Extrapolation of Imaging Data to Vesicle Population Distribution

To reconstruct distributions of real vesicle diameters based on our imaged sections, we used a mathematical model based on weighted contributions of randomly sectioned coexisting subpopulations of spherical objects.

**Figure 1.**
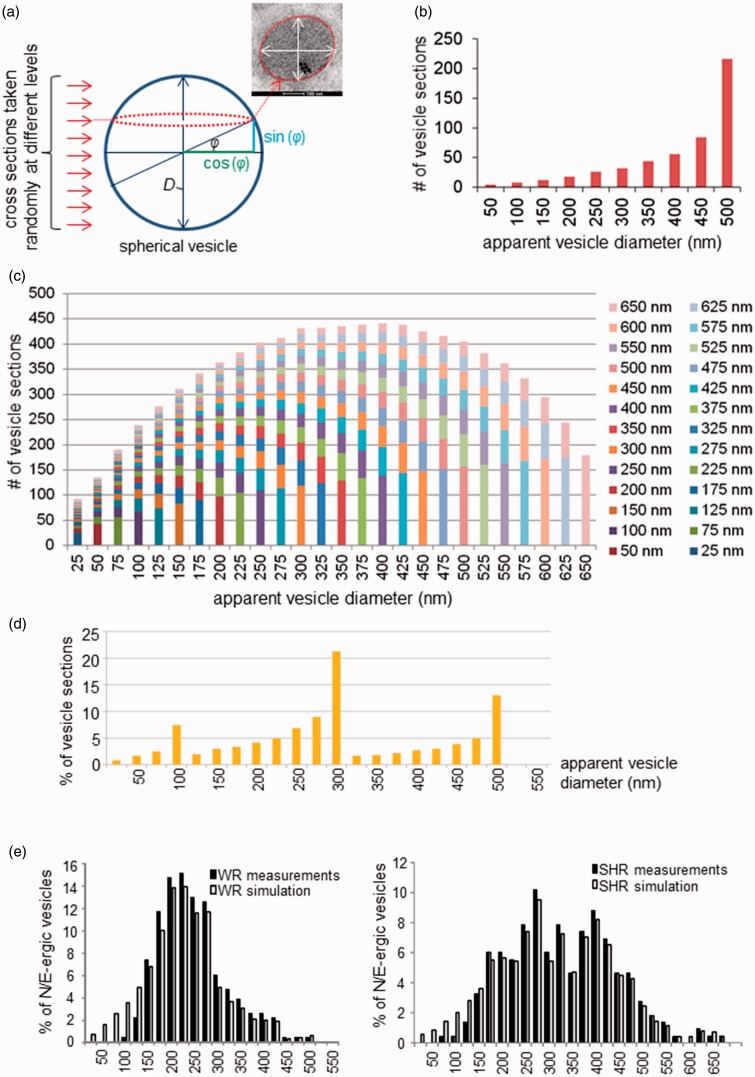
Extrapolation of imaging data to *true* vesicle population distribution by a mathematical model which corrects for eccentric optical imaging. (a) Geometrical basis for the mathematical model describing the distribution of optical sections randomly taken from vesicles with diameter *D*: Only optical sectioning through the center reveals the true *D*. Random sectioning at a distance of sin (φ)* × D* from the center yields diameters corresponding to cos (φ)* × D*. (b) Distribution of 500 optical sections (apparent vesicle diameters) taken randomly from a uniform population of vesicles with *D* = 500 nm. (c) Distribution of optical sections (apparent vesicle diameters) taken randomly from coexisting, equally contributing, subpopulations of vesicles of diameters ranging from 25 to 650 nm. This distribution serves as basis for simulated data: By adjusting the relative contributions of different vesicle size groups, the overall shape of the histogram can be developed to match experimental data. (d) Simulation of the normalized distribution of optical sections (apparent vesicle diameters) taken randomly from coexisting vesicle populations measuring diameters of 100 nm, 300 nm, and 500 nm and represented in a ratio of 1:2:1, respectively. (e) Normalized distributions of noradrenergic and adrenergic vesicle diameters in WR (left) and SHR (right). Black bars—Electron microscopic measurements as shown in Results section (Figure 5(a)); white bars—simulated vesicles diameters based on a randomly sectioned true vesicle population of assorted sizes. The contribution of each size group of true vesicles was weighted to achieve a high correlation between data and simulation by linear regression (WR: *R*^2^ = .97; SHR: *R*^2^ = .99). SHR = spontaneously hypertensive rats; WR = Wistar rats.

Sectioning of a sphere-like object results in discs of a range of diameters, from 0 at its base and apex, to the true diameter through the center of the sphere (Figure 1(a)). In a more complex setting, if multiple spheres of different sizes are sectioned simultaneously, any but the largest resulting disc may stem from a smaller sphere, cut closer to its center, or from a larger sphere, cut at a level more distant from its center. EM image analysis yields cross sections of such a heterogeneous population of sphere-like vesicles at random levels, occasionally including sections through their largest diameter at their centers. Therefore, analysis of vesicle sections underestimates the *true* vesicle diameters and skews the size distributions leftward (Figure 1(b)).

To estimate the real vesicle diameter distribution in our study, we modelled the overall vesicle size distribution using the weighted contributions of coexisting vesicle subpopulations, optimizing the weighting by least-squares linear regression to fit the observed data. With equal probability of optical sectioning of a vesicle at any level, from base through center to apex, we assumed equal distribution of sin (φ) between −1 and +1 (Figure 1(a)). The resulting diameters of each section then correspond to 2 *×* cos (φ) scaled to the true vesicle diameter (*D*) as obtained through sectioning the vesicle center. For any given vesicle population with a uniform diameter, EM imaging is therefore predicted to yield a distribution of optical cross sections, which is negatively skewed with a maximum bin corresponding to the true vesicle diameter (Figure 1(b)). We used a template which assumes that all vesicles are optically sectioned with equal probability at 1-nm distances and which incorporates vesicle groups from 0 to 700 nm, in 25-nm-wide bins (Figure 1(b) to (e)). By weighting the contributions of each vesicle size group, we simulated a distribution that correlated well (linear regression *R*^2^ > .95) with our raw data (Figure 1(e)). The solution of the problem closely resembles the approach described by Wicksell (1925) and is applicable to a size range of below one order of magnitude as in the present study. This simple approach may be easily followed using widely available spreadsheet software (e.g., Microsoft Excel; see Online Supplemental Material).

## Results

### DbH and NPY Expression and Coexpression at Light Microscope (Cellular) Level

**Figure 2.**
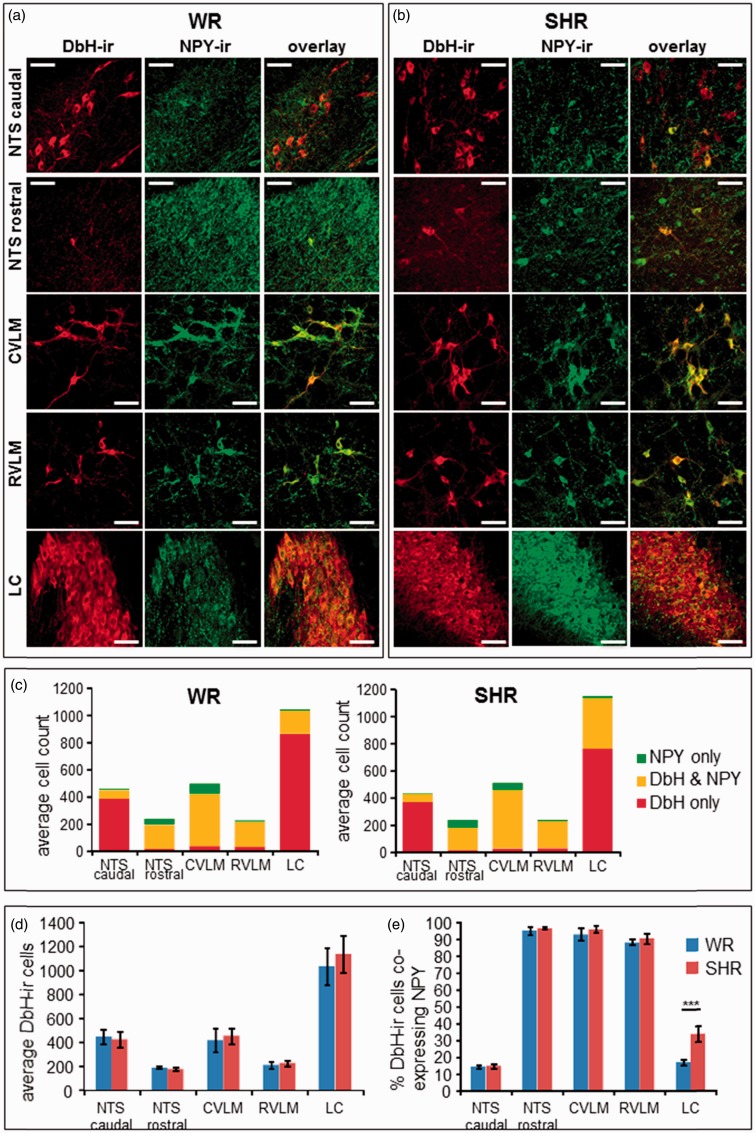
DbH and NPY expression in brain areas containing N/E-ergic cell groups. (a) and (b) Representative confocal images of (DbH-ir (in red; left columns) and NPY-ir (in green; middle columns) at the level of caudal and rostral NTS, CVLM, RVLM, and LC in WR (a) and SHR (b). The overlaid red/green images (right hand columns) show the degree of colocalization (in yellow). Note the high density of NPY-positive but DbH-negative fibers and varicosities in the NTS and the LC in both rat strains. Scale bars 50 μm. (c) Average counts of cell bodies with DbH-ir only (red), NPY-ir only (green), and DbH–NPY colocalization (yellow) across the N/E-ergic brain areas in WR (left panel; *n* = 3) and SHR (right panel; *n* = 3). (d) Comparison of average numbers of DbH-ir cell bodies between WR (blue) and SHR (red) shows no significant differences between strains (*p* > .25, Student's unpaired *t* test, *n* = 3). (e) Comparison of the fraction of DbH-ir cells which coexpress NPY across N/E-ergic cell groups. A significant difference between WR (blue) and SHR (red) was found in the LC (***p < .01, Student's unpaired *t* test, *n* = 3 rats). CVLM = caudal ventrolateral medulla; DbH = dopamine-β-hydroxylase; DbH-ir = DbH-immunoreactivity; LC = locus coeruleus; NPY = neuropeptide Y; NPY-ir = NPY-immunoreactivity; NTS = nucleus tractus solitarii; RVLM = rostral ventrolateral medulla; SHR = spontaneously hypertensive rats; WR = Wistar rats.

Confocal imaging of WR as well as SHR brain sections revealed that the majority of neuropeptide Y releasing (NPY-expressing) neurons across all investigated N/E-ergic cell groups coexpress DbH. Viewed from the other perspective, the majority of N/E-ergic cell bodies in rostral NTS (A2 neurons), caudal ventrolateral medulla (A1 neurons), and RVLM (C1 neurones) coexpressed NPY (Figure 2(a) to (c), (e)). In contrast, the caudal part of the NTS contained more N/E-ergic and fewer NPY-ergic cells as compared with the rostral NTS (Figure 2(c)), resulting in a lower degree of colocalization in both rat strains (caudal 14.0 ± 1.0% versus rostral 95.1 ± 2.4% for WR; 14.4 ± 1.2% vs. 96.4 ± 0.8% for SHR; Figure 2(e)).

In the LC, the area with the highest density of N/E-ergic neurons, only partial colocalization of N/E with NPY was found (Figure 2(c)). Here, a significant difference was observed between WR and SHR. Although the number of DbH-expressing cells was similar (Figure 2(d)), NPY-positive cells were much more abundant in SHR (34.3 ± 5.0% vs. 17.1 ± 1.6% in WR; *p* < .001), leading to a higher degree of DbH-NPY colocalization (33.5 ± 4.7% in SHR vs. 16.6 ± 1.8% in WR; *p* = .015; Figure 2).

### Evidence of Preservation of Ultrastructure and Antigenicity of N/E-ergic Vesicles in EM Samples

**Figure 3.**
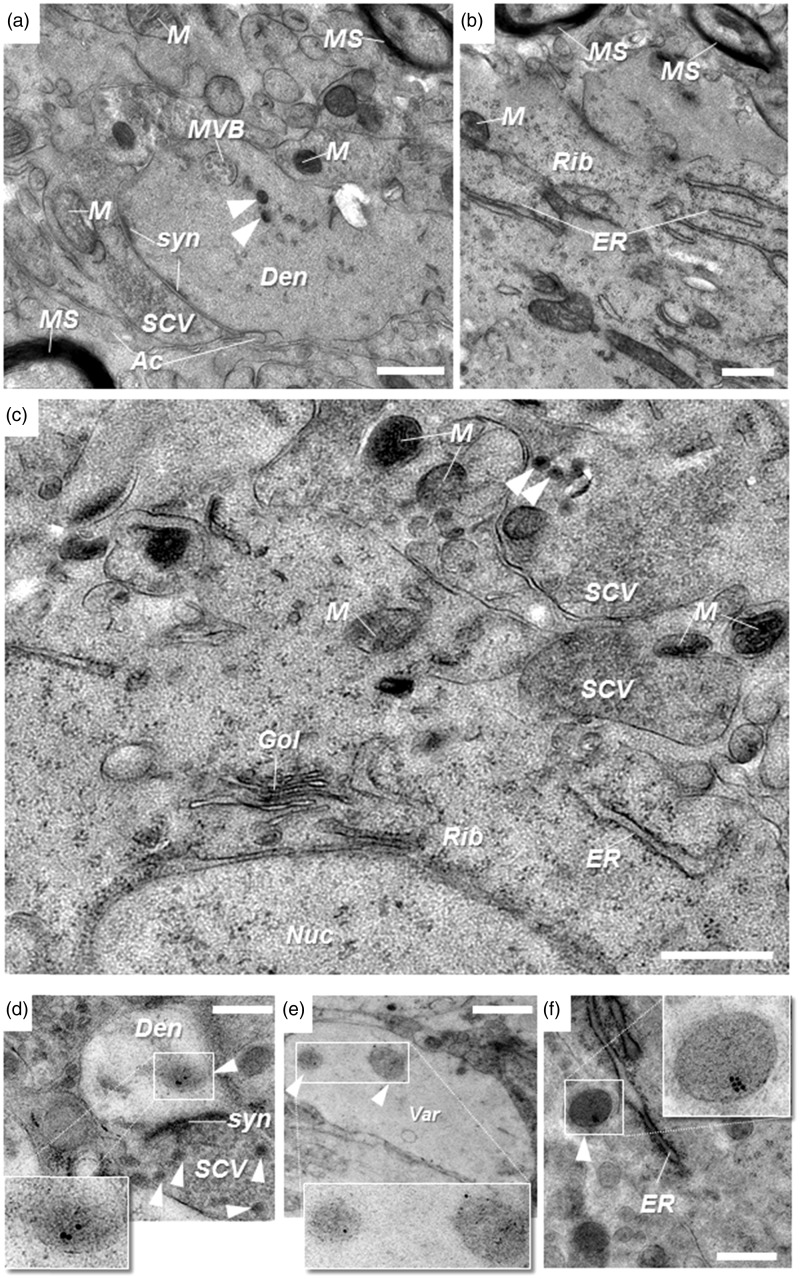
Preservation of brain tissue ultrastructure, including antigenicity of noradrenergic and adrenergic markers, using cryo-fixation, freeze-substitution, and Lowicryl resin embedding. Representative electron microscopic images from nucleus tractus solitarii ((a) and (d)), rostral ventrolateral medulla ((b) and (e)), and locus coeruleus ((c) and (f)) regions. Note the structural integrity of nuclear, mitochondrial, and plasma membranes, and the delineation of vesicular, Golgi, and ER membranes, as well as MS. (d)–(f) Immuno-gold labeling (10 nm) for dopamine-β-hydroxylase ((d) and (f)) and vesicular monoamine transporter 2 (e) identifies N/E-ergic large dense core vesicles (in insets). White arrowheads denote dense core vesicles (100–500 nm). *Ac = *putative astroglial process; Den = putative dendrite; *ER* = endoplasmic reticulum; *Gol* = Golgi; *M* = mitochondrion; *MS* = myelin sheath; *MVB* = multivesicular body; *Nuc* = nucleus; *Rib* = area rich in ribosomes; *SCV* = area rich in small clear vesicles; *syn* = synaptic contact; *Var* = putative N/E-ergic varicosity. Scale bars 500 nm.

**Figure 4.**
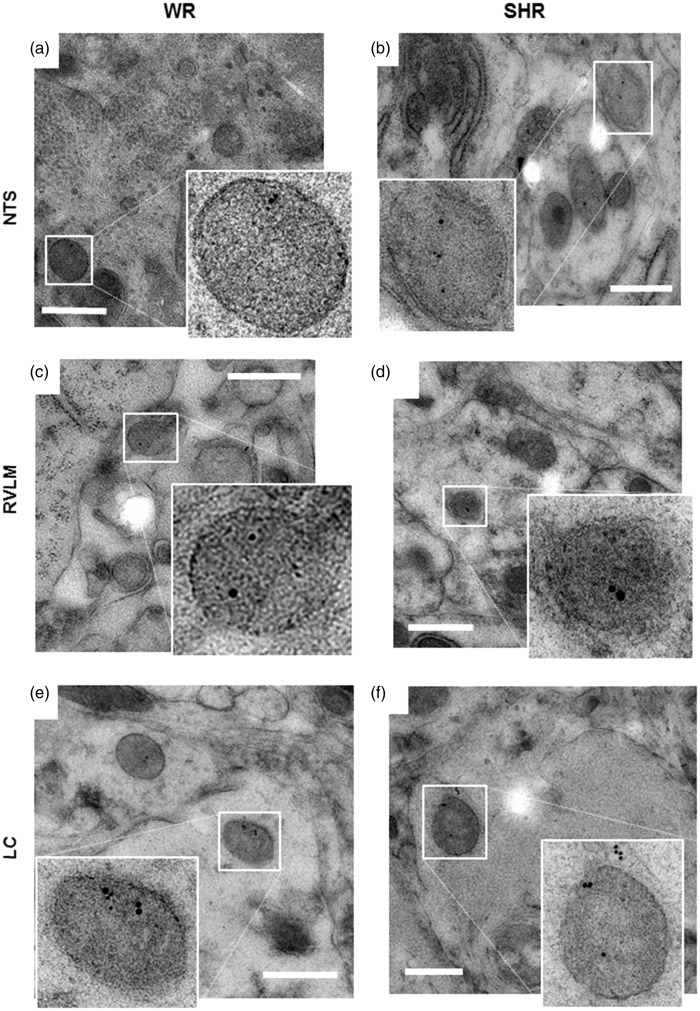
Representative electron microscopic images of large vesicles with norepinephrine and epinephrine and neuropeptide Y (NPY) colocalization. Double immuno-gold labeling for dopamine-β-hydroxylase or vesicular monoamine transporter 2 (10 nm gold) and NPY (15 nm gold) in nucleus tractus solitarii ((a) and (b)), RVLM ((c) and (d)), and LC ((e) and (f)) identifies large dense core vesicles in the 250 to 500 nm diameter range in adult rat brain of WR ((a), (c), and (e)) and SHR ((b), (d), and (e)), which costore NPY with norepinephrine and epinephrine. Scale bars are 500 nm. Inset shows selected double-labeled LDCV. LC = locus coeruleus; RVLM = rostral ventrolateral medulla; SHR = spontaneously hypertensive rat; WR = Wistar rat.

The EM approach employed in this study was optimized for the best compromise between preservation of ultrastructure and antigenicity of tissue from NTS, RVLM, and LC. Dehydration issues appeared to be minimal, based on the insignificant cytosolic extraction, and freezing artifacts were also, in general, absent as indicated by the intact appearance of nuclei, the delineation and structural integrity of the nuclear, vesicular, and plasma membranes, and preservation of myelin sheaths (Figure 3; Brown et al., 2009). Organelles such as the endoplasmic reticulum, ribosomes, Golgi, mitochondria, multivesicular bodies, and individual synapses were easily recognized (Figure 3). Small clear vesicles sections of 40 to 60 nm diameter and LDCV sections of over 80 nm and varying electron opacity were clearly identifiable (Figure 3). LDCV were spherical or slightly ellipsoid, with a trend to deviation from spherical shape in the larger vesicles (Figure 3(d) to (f)). Antigenicity was well maintained as confirmed by immunolabeling with colloidal gold particles for DbH and VMAT2 (10 nm gold) and NPY (15 nm gold); small clear vesicles and most dense core vesicles of diameters under 120 nm were unlabeled (Figures 3 and 4). Labeled LDCV were found in perikarya, as well as putative dendrites, putative varicosities, and axonal processes. In cell bodies, they were located in peripheral portions of cytoplasm and in dendritic and axonal compartments, typically remote from any synapse-like structures (Figures 3 and 4).

### Comparative EM Analysis of N/E-ergic Vesicles in WR and SHR

**Figure 5.**
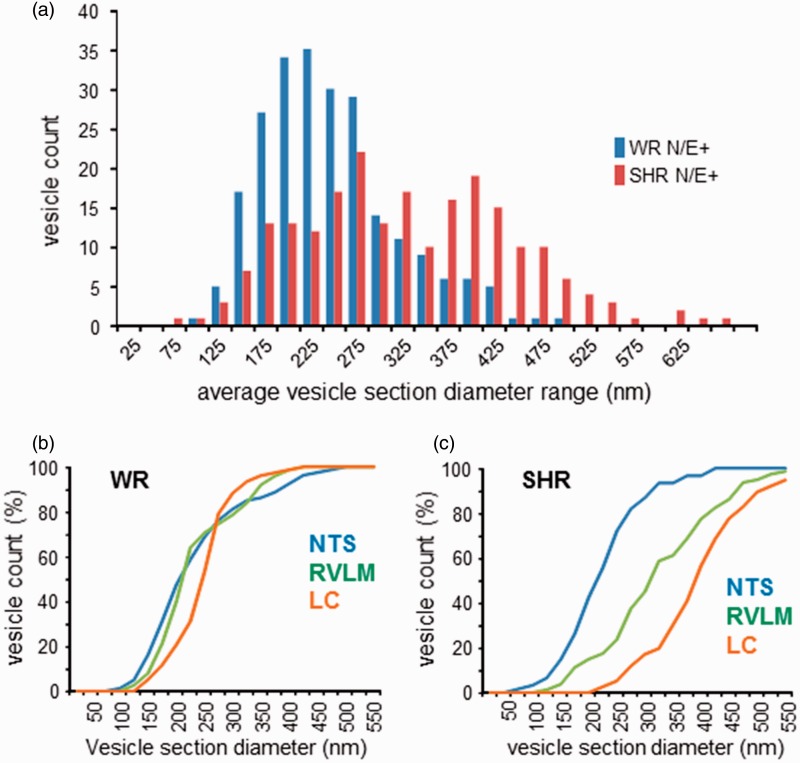
Electron microscopic analysis of noradrenergic and adrenergic (N/E-ergic) vesicles in WR and SHR. (a) Distribution of vesicles positively labeled for N/E-ergic markers across three areas—NTS, RVLM, and LC (WR: *n* = 232; SHR: *n* = 217). N/E-ergic vesicle sections were larger in SHR. (b) In WR, normalized cumulative histograms of large dense core vesicles (LDCV) section diameters showed similar distributions in NTS (blue; mean 230 nm, median 205 nm, *n* = 80) and RVLM (green, mean 231 nm, median 212 nm, *n* = 75; *p* = .384, two-tailed Kolmogorov–Smirnov [KS] test). The LC distribution (orange, mean 245 nm, median 249 nm, *n* = 77) differed significantly from the other two areas (*p* ≤ .001, two-tailed KS test). (b) In SHR, the (normalized) distributions of LDCV section diameters in NTS (blue; mean 219 nm, median 212 nm, *n* = 61), in RVLM (green, mean 318 nm, median 304 nm, *n* = 80), and in LC (orange, mean 393 nm, median 391 nm, *n* = 76) were all significantly different from one another (*p* ≤ .001, two-tailed KS test). LC = locus coeruleus; NTS = nucleus tractus solitarii; RVLM = rostral ventrolateral medulla; SHR = spontaneously hypertensive rat; WR = Wistar rat.

Across all N/E-ergic brain areas imaged in this study, labeled LDCV sections measured diameters in the range of 70 to 650 nm, with only 2.5% of the population smaller than 120 nm (Figures 4 and 5(a)). In WR, the distribution of LDCV diameters peaked over the 200 to 225 nm range and was skewed toward the right, with mean and median of 235 and 223 nm, respectively (Figure 5(a)). In SHR, the distribution of N/E-ergic LDCV was shifted to the right and appeared bimodal, with peaks in 250 to 275 nm and the 375 to 400 nm ranges; the mean diameter overall was 317 nm and the median 306 nm (Figure 5(a)). These measurements are consistent with markedly larger sizes of central N/E-ergic LDCV than had been reported by earlier studies.

Detailed analysis revealed significant area-specific differences in LDCV section sizes. While NTS and RVLM populations in WR were not significantly different from one another and were strongly right skewed, both differed significantly from the more normally distributed LC population (Figure 5(b)). In SHR, the RVLM contained a population of N/E-ergic LDCV, which was significantly larger than that in NTS but significantly smaller than that found in LC (Figure 5(c)).

**Figure 6.**
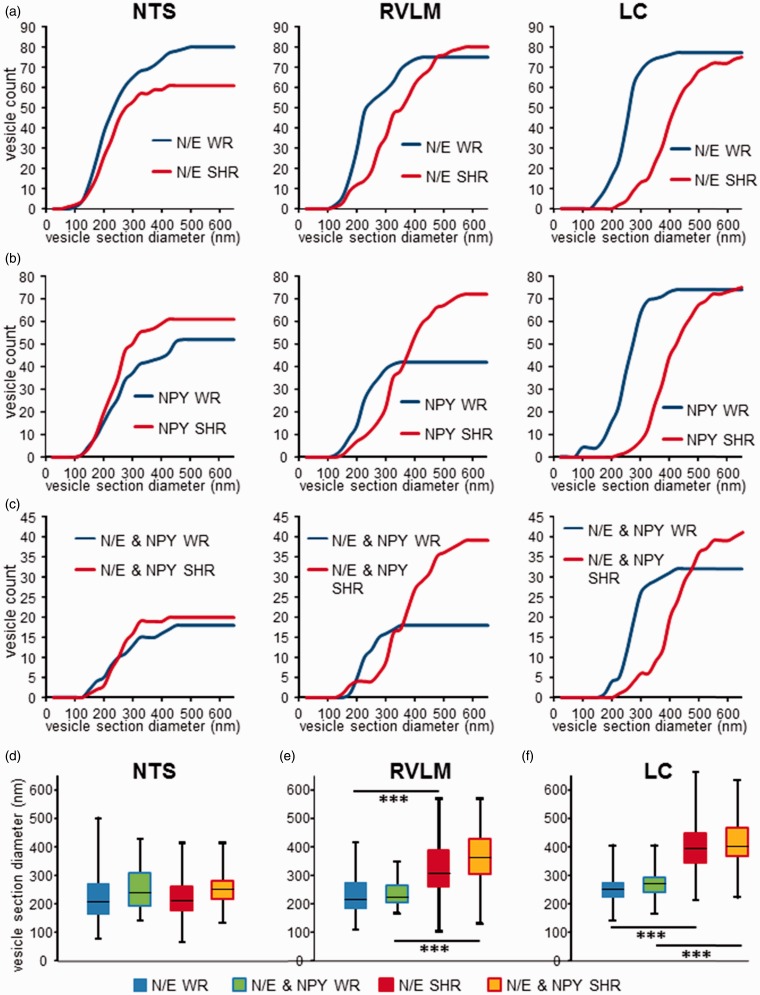
Electron microscopic analysis of colocalization of N/E and NPY in vesicles in WR and SHR. (a) Cumulative distributions of diameters of large dense core vesicles (LDCV) labeled with noradrenergic and adrenergic (N/E-ergic) markers were not different between WR (blue) and SHR (red) in the NTS (left panel; *p* = .799; *n*_WR_ = 80, *n*_SHR_ = 61) but significantly different in RVLM (middle panel; *p* ≤ .001; *n*_WR_ = 75, *n*_SHR_ = 80) and in LC (right panel; *p* ≤ .001; *n*_WR_ = 77, *n*_SHR_ = 76; two-tailed KS test). (b) Cumulative distributions of diameters of NPY-labeled LDCV were not different between WR (blue) and SHR (red) in the NTS (left panel; *p* = .390; *n*_WR_ = 52, *n*_SHR_ = 61) but significantly right shifted in RVLM (middle panel; *p* ≤ .001; *n*_WR_ = 42, *n*_SHR_ = 72) and further right shifted in LC (right panel; *p* ≤ .001; *n*_WR_ = 71, *n*_SHR_ = 75; two-tailed Kolmogorov–Smirnov [KS] test). (c) Cumulative distributions of diameters of LDCV sections colabeled for NPY and a N/E marker were similarly distributed as N/E-ergic (a) and NPY-ergic (b) sections in their corresponding brain areas. Again, no difference between WR and SHR was detected in the NTS (left panel; *p* = .850; *n*_WR_ = 18, *n*_SHR_ = 20) but distribution in RVLM (*n*_WR_ = 18, *n*_SHR_ = 39) and LC (*n*_WR_ = 32, *n*_SHR_ = 41) differed significantly between strains (middle and right panels; *p* ≤ .001, two-tailed KS test). (d) to (f) Box-and-whisker diagrams of N/E-ergic (WR: blue; SHR: red) and N/E–NPY-colocalizing (WR: yellow/blue; SHR: yellow/red) vesicle diameters for NTS (d), RVLM (e), and LC (f). Boxes represent first to third quartile, black horizontal bars represent median of data, and whiskers indicate data range. ***p ≤ .001, two-tailed KS test. LC = locus coeruleus; N/E = norepinephrine and epinephrine; NTS = nucleus tractus solitarii; RVLM = rostral ventrolateral medulla; SHR = spontaneously hypertensive rat; WR = Wistar rat.

In the NTS, N/E-ergic vesicle populations were not different between WR and SHR (Figure 6(a), left and 6(d)). However, in RVLM as well as LC, SHR tissue contained larger vesicles (Figure 6(a), middle and right, (e), and (f)). The distribution of diameters of NPY-positive LDCV was not significantly different to what was found for N/E-labeled LDCV in the corresponding N/E-ergic sections (Figure 6(a) and (b)). However, the number of NPY-positive vesicles appeared higher in the RVLM of SHR as compared with WR. Thus, the significant differences between WR and SHR in RVLM and LC, as described earlier for N/E-ergic LDCV, also held for NPY-labeled vesicle sections (Figure 6(b)) and for the subpopulation of vesicles colabeled for N/E and NPY (Figure 6(c) to (f)). These data suggest a significant increase in N/E-ergic vesicle size and in the frequency of colocalization with NPY in RVLM and LC of SHR.

### Reconstruction of Vesicle Size Distributions by Correction for Eccentric Optical Sectioning and Estimation of Intravesicular N/E Concentration

Sectioning of cell organelles occurs at random and therefore diameter measurements based on EM images of the vesicles will underestimate their true diameters and volumes. Using our mathematical model (see Methods section, Figure 1), we simulated the distribution of the true diameters of vesicles from which the EM image measurements were taken. The model weights the contribution of differently sized vesicle groups to the total population and predicts the resulting distribution of optical sections. We assumed a mixed population of different sized vesicles and weighted each size group individually to optimize the correlation between EM data distribution curves and simulated data.

**Figure 7.**
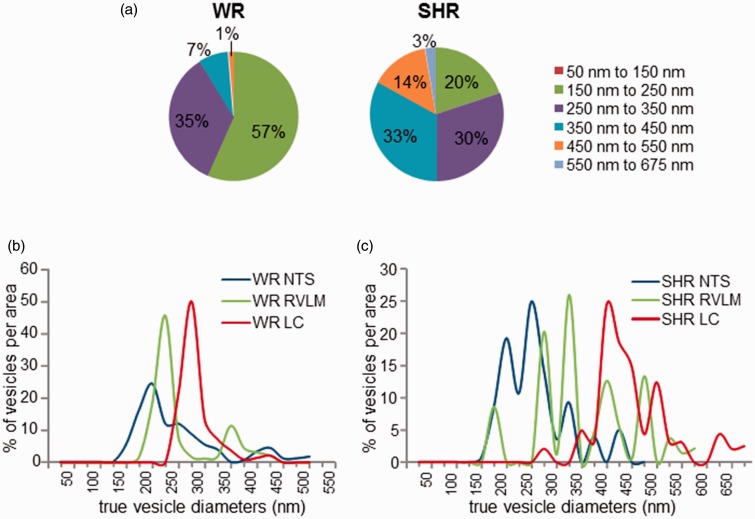
Mathematical modeling and reconstruction of *true* vesicle size distributions corrected for off-centered optical sectioning. (a) Contribution of diameter bands of up to 150 nm, 250 nm, 350 nm, and so on, to the entire true vesicle population in WR (left) and SHR (right). (b) Distribution of true vesicle diameters after correction for random optical sampling for different noradrenergic and adrenergic (N/E-ergic) areas (NTS: blue; RVLM: green; LC: red) in WR shows bimodal distributions for NTS and RVLM and a unimodal range in LC. (c) Modeling of of true vesicle diameters in SHR for different N/E-ergic areas (NTS: blue; RVLM: green; LC: red) shows broader distributions with multiple peaks in RVLM particularly. Populations are shifted to larger diameters for RVLM and LC as compared with WR. LC = locus coeruleus; NTS = nucleus tractus solitarii; RVLM = rostral ventrolateral medulla; SHR = spontaneously hypertensive rat; WR = Wistar rats.

This analysis suggested that across the three brain areas investigated, in WR, 57% of labeled N/E-ergic vesicles were in the 150 to 250 nm diameter range, 35% measured between 250 and 350 nm, and 8% belonged to a population of over 350 nm and up to 550 nm (Figure 7(a)).

In SHR, only 20% of N/E-ergic vesicles belonged to the 150 to 250 nm diameter range, 30% were between 250 and 350 nm in diameter, 33% belonged to the 350 to 450 nm range, and 17% were even larger than 450 nm (Figure 7(a)). Therefore, large vesicles were much more abundant in SHR.

When applying the regression model to the different N/E-ergic brain areas separately, the resulting true vesicle distributions retained the overall shape of the vesicle image distributions (Figure 5), but, as expected, the peaks were slightly right shifted as vesicles were now represented by their maximal diameters rather than off-centered, smaller cross sections. Smaller diameter (<150 nm) vesicles were no longer represented in the true distributions (Figure 7(a) and (b)). In WR, major population peaks became apparent at 175 to 200 nm in NTS, 200 to 225 nm in RVLM, and 250 to 275 nm in LC, with additional minor peaks for NTS at 400 to 425 nm and 325 to 350 nm for RVLM (Figure 7(b)). Distributions in SHR were not only shifted to larger diameter ranges but were also broader and showed multiple peaks (Figure 7(c)).

These observations consistently suggest that, in the SHR, there is area-specific reorganization of catecholamine packaging in a way, which would favor release of large transmitter quantities upon individual fusion events.

## Discussion

In this study, we combined EM with confocal imaging and mathematical modeling to characterize central N/E-containing vesicles at a new level of precision. Our new data help to explain how central N/E neurons operate in the volume transmission mode. We also mapped central coexpression and colocalization of N/E and NPY at cellular and vesicular levels. To investigate the cross-links between central N/E-ergic and NPY-ergic transmission, control of blood pressure, and modulation of impulsivity, we compared these transmitter systems between WR and SHR, a genetic rat model with altered catecholaminergic transmission.

At cellular level, we found considerable but area-specific overlap between N/E-ergic and NPY-ergic systems and differences in colocalization in the LC of the SHR. At the ultrastructural level, we established a clear morphological correlate for the previously detected extra large quantal N/E release events and revealed that in SHR, there are many more large LDCV in the presympathetic C1 area and in the A6 group, which is likely to affect the physiological consequences and cellular targets of these central modulator systems. This analysis has been made possible by the combination of the advanced cryo-preservation methods used here with the greatly increased probability of locating N/E processes in the tissue by fluorescence microscopy prescreening.

Remarkably, we found specifically labeled N/E-ergic vesicles with diameters above 150 nm and up to 650 nm with population peaks around 200 to 300 nm and 250 to 400 nm for WR and SHR, respectively. This size range is considerably larger than documented by previous literature for the brain, although comparable to adrenal chromaffin granules, and consistent with volume transmission, particularly as their location was invariably remote from synapse-like structures. Quantal content of catecholaminergic vesicles is related to their volume (Colliver et al., 2000), and large quanta, once released, are more likely to saturate local transport/reuptake mechanisms and allow farther diffusion of N/E to reach more remote and a wider range of target cells. Previous work from our own group for the first time described central exocytotic N/E release and measured quantal sizes using amperometry in explants from rat brainstem of young rats (Chiti and Teschemacher, 2007; Teschemacher et al., 2008). The majority of exocytotic events released around 10^5^ molecules of N/E per quantum but larger events ranging up to 10^7^ N/E molecules were detected. If we assume similar vesicle anatomy in young and adult rats (as used in the present study), our current findings put us in the position to estimate vesicular volumes, correlate them with quantal size, and thus suggest intravesicular N/E concentrations of around 10 mM in NTS and RVLM.

Earlier studies appear to have overlooked large-sized vesicle populations, potentially due to use of detergents and resulting diffuse reaction products that may have limited the spatial resolution and accurate distinction between adjacent vesicles. On the other hand, we may, to some extent, have underestimated and biased against the populations below 150 nm in diameter because we optimized our labeling procedure to enhance specificity at the cost of labeling density. Indeed, when comparing vesicle imaging data with simulated true LDCV diameters, actual detected N/E-ergic vesicles were found less frequent in the lower range of bins up to 100 nm than our model predicted (Figure 1(e)).

Despite the obvious similarities, such as their overall cellular morphology and their ability to synthesize catecholamines, during embryogenesis, the LC is derived from the metencephalon, while the lateral tegmental system, which gives rise to NTS and RVLM, develops from the myelencephalon. Importantly, C1, A2, and A6 N/E-ergic groups (in RVLM, NTS, and LC, respectively) are involved in very different brain functions. On the one hand, these differences are reflected by their distinct central connectivity, but equally, it may be underpinned by differences in specific mechanisms of N/E storage, cotransmitter content, and exocytotic release. We hypothesize that changes in these parameters may correlate with described functional deficits in the SHR. A potential driver for vesicle enlargement in SHR may be the upregulated expression of intravesicular N/E-chelating proteins such as chromogranin A (O'Connor et al., 1999).

Interestingly, N/E-ergic vesicles in LC were larger than in other inspected areas in both, WR and SHR, but in SHR the shift toward larger sizes was particularly dramatic. A parallel shift to larger diameters was seen for NPY-labeled vesicles in SHR. The fraction of N/E-NPY colocalizing vesicles was increased from 41% in WR to 54% in SHR, whereby in both rat strains, the NPY-colabeling was found in the larger of N/E-ergic vesicles. While it is still under debate whether the symptoms of ADHD represent hyper- or hypofunction of the central N/E-ergic system, it is generally accepted that an optimum N/E-ergic tone in the prefrontal cortex is required for adequate executive function, where release must be counterbalanced by uptake transporter activity (Russell and Wiggins, 2000; Heal et al., 2008; Prince, 2008; Howells et al., 2012). Conversely, an unbalanced NPY-ergic system has been implicated as potential causative factor in ADHD (Lesch et al., 2011; Bari et al., 2015), and significant changes in NPY expression levels across different brain areas have been reported in SHR (Maccarrone and Jarrott, 1985). Our data are consistent with N/E-ergic and NPY-ergic hyperactivity and increased turnover in the forebrain where N/E-ergic axons from the LC project (Russell, 2002; Imeraj et al., 2012).

The other area where significantly larger vesicles containing N/E, NPY, or both transmitters were detected in SHR was the presympathetic C1 group in the RVLM. Here, the fraction of costoring vesicles doubled (from 24% to 49%). Overall expression and coexpression of N/E and NPY in the RVLM were, however, not different at the light microscopic level, indicating that the same neuronal population has increased transmitter levels and cotransmission in the hypertensive strain. Importantly, the increased vesicle sizes in SHR are in agreement with larger quantal sizes, which we observed in amperometric recordings from prehypertensive RVLM tissue (Teschemacher et al., 2008). This, in turn, is expected to result in an increase of the impact and range of signaling in SHR and may anatomically underpin the prohypertensive function of C1 activity as a potential driver of neurogenic hypertension (Colombari et al., 2001; Kasparov and Teschemacher, 2008; Abbott et al., 2012).

In the NTS, at cellular resolution, no significant differences in numbers of neurons expressing N/E or NPY, nor in N/E-NPY colocalization levels were observed between WR and SHR. This lack of difference between rat strains was reflected in similar size distributions of N/E-ergic and NPY-ergic vesicles, and an only modest increase in colocalization of both transmitters in N/E-ergic vesicles from 24% in WR to 32% in SHR. Therefore, for A2 neurons in the NTS, the morphological evidence is also in agreement with earlier amperometric analysis from our group, which had suggested a comparable range of quantal N/E-ergic release events in young prehypertensive WR and SHR (Teschemacher et al., 2008).

While a large body of previous work and our own study provide clear functional links between signaling mediated by N/E and NPY, the exact nature of this interaction is still elusive. As NPY activates several types of G_i_-protein-coupled receptors which may be located pre- or postsynaptically with respect to N/E-ergic neurons or their axonal projections, predictably, the effects of cotransmission are complex and area specific (Gehlert, 2004). In addition, nonneuronal brain cells must be taken into account as, according to recently published transcriptomes of mouse forebrain, astrocytes express predominantly β1-, and also α1- and α2-adrenoceptors and Y1 receptors at lower levels (Cahoy et al., 2008; Zhang et al., 2014). Clues on the functional impact of NPY cotransmission can be gained from studies that have applied NPY into N/E-ergic brain areas or sites receiving N/E-ergic input. For example, in the LC, Y2 receptor activation enhances N/E-ergic signaling and is implicated in anxiolytic actions, while Y1 receptors appear to counteract Y2 actions (Illes et al., 1993; Kask et al., 1998). Regarding cardiovascular regulation, NPY within the RVLM as well as NTS was shown to modulate blood pressure differentially via antagonistic actions of Y1 and α2-adrenoceptors or of Y1 and Y2 receptors (Diaz-Cabiale et al., 2007). Interestingly, similar to N/E, NPY also acts on astrocytes where it induces glycogenolysis (Sorg and Magistretti, 1991), which will inevitably lead to release of l-lactate. The recent discovery of a signaling role for l-lactate in the brain possibly opens a new avenue here, as it seems feasible that the larger vesicles in SHR with higher NPY content would allow more effective recruitment of astrocytes by chemical signals released by N/E-ergic cells (Tang et al., 2014). We believe that this interaction needs to be studied further in order to clarify how a shift in the vesicular content of N/E or NPY affects the signaling between N/E-ergic fibers and their downstream targets.

In summary, we have performed extensive quantitative analysis of central N/E-containing vesicles and colocalization between N/E and NPY at light and EM level. This has allowed us to draw a numerically representative comparison between WR and SHR, a widely used model for essential hypertension and ADHD. Changes which were detected in the SHR are consistent with the notion of altered catecholaminergic signaling and suggest that area-specific aberrant transmitter release mechanisms reflect the specific neurological and behavioral phenotype of SHR.

## Summary

In an animal model for hypertension and attention-defcit/hyperactivity disorder, the storage compartments containing adrenaline-like signaling molecules were significantly larger in specific brain areas and contained more of the chemical comessenger, neuropeptide Y, suggesting an extended and altered signaling range.

## Supplementary Material

Supplementary material
